# Serum hsCRP in early pregnancy and preterm delivery in twin gestations: a prospective cohort study

**DOI:** 10.1186/s12884-023-05445-4

**Published:** 2023-02-21

**Authors:** Yang-yang Chen, Yu-bo Zhou, Jing Yang, Yu-meng Hua, Peng-bo Yuan, Ai-ping Liu, Yuan Wei

**Affiliations:** 1 Department of Social Medicine and Health Education, School of Public Health, Peking University, 38 Xueyuan Road, Haidian District, Beijing, 100191 China; 2 Institute of Reproductive and Child Health, National Health Commission Key Laboratory of Reproductive Health, Peking University, Beijing, 100191 China; 3 Department of Epidemiology and Biostatistics, School of Public Health, Peking University, Beijing, 100191 China; 4 Department of Obstetrics and Gynecology, Peking University Third Hospital, Beijing, 100191 China

**Keywords:** CRP, Inflammation, Preterm delivery, Twin gestations, Early pregnancy

## Abstract

**Background:**

Systemic inflammation during pregnancy may be associated with preterm delivery (PTD), but data for twin gestations are lacking. The aim of this study was to examine the association of serum high-sensitivity C-reactive protein (hsCRP), a marker of inflammation, in early pregnancy of twin gestations with risk of PTD, including spontaneous (sPTD) and medical-induced preterm delivery (mPTD).

**Methods:**

A prospective cohort study involved 618 twin gestations was conducted in a tertiary hospital in Beijing, from 2017 to 2020. Serum samples collected in early pregnancy were analyzed for hsCRP using particle-enhanced immunoturbidimetric method. Unadjusted and adjusted geometric means (GM) of hsCRP were estimated using linear regression, and compared between PTD before 37 weeks of gestation and term delivery at 37 or more weeks of gestation using Mann–Whitney rank sum test. The association between hsCRP tertiles and PTDs was estimated using logistic regression, and further converted overestimated odds ratios into relative risks (RR).

**Results:**

A total of 302 (48.87%) women were classified as PTD, with 166 sPTD and 136 mPTD. The adjusted GM of serum hsCRP was higher in PTDs (2.13 mg/L, 95% confidence interval [CI] 2.09 –2.16) compared to term deliveries (1.84 mg/L, 95% CI 1.80 –1.88) (*P* < 0.001). Compared with the lowest tertile of hsCRP, the highest tertile was associated with increased risk of PTD (adjusted relative risks [ARR] 1.42; 95% CI: 1.08–1.78). Among twin pregnancies, the adjusted association between high values of serum hsCRP in early pregnancy and preterm delivery was only observed in the subgroup of spontaneous preterm deliveries (ARR 1.49, 95%CI:1.08–1.93).

**Conclusions:**

Elevated hsCRP in early pregnancy was associated with increased risk of PTD, particular the risk of sPTD in twin gestations.

## Background

High infant morbidity and mortality in twin gestations are predominantly related to preterm delivery (PTD) [1, 2]. Among twin pregnancies, the risk of PTD is up to 56.6% [3], which still rising in most nations [4, 5], making a major concern in modern obstetrics.

PTD can be categorized into three subtypes including spontaneous PTD (sPTD), preterm premature rupture of membranes (PROM), and medical-induced PTD (mPTD) [4]. PROM, as a precursor, is commonly incorporated into sPTD [6]. sPTD accounts for 60–70% of PTD, and the remaining 30–40% was mPTD [4, 7]. sPTD is considered as a syndrome caused by multiple mechanisms such as inflammation and uteroplacental ischaemia [7], and common indications for mPTD include preeclampsia and fetal stress, et al. [8]. Additionally, for twin gestations, monochorionic pregnancy is recommended to be electively induced ≤ 36 weeks of gestation to decrease the potential risks [9].

Systemic inflammation stimulates the secretion of cytokines from the pregnant tissues and further the action in cascades, contributing to multiple pathways of sPTD [4, 7]. C-reactive protein (CRP) is a typical biomarker in response to inflammatory stimulation [10]. And high-sensitive CRP (hsCRP), with lower limit of detection, can reflect low-grade inflammation. Previous studies in singleton indicated that elevated maternal serum CRP or hsCRP was associated with increased risk of sPTD [11–13]. However, related data is lacking for twins. Only two studies on twins reported a positive association of Interleukin-8, another inflammatory indicator, in mid-pregnancy with sPTD [14, 15].

The objective of this prospective cohort study was to test the hypothesis that hsCRP in early pregnancy may increase the risk of PTD in twin gestations.

## Methods

### Participants and data collection

A prospective multicenter University Hospital Advanced Age Pregnant (UNIHOPE) cohort was conducted from 2017 to 2020 in China [16]. In the study, data on twin gestations collected in a tertiary hospital in Beijing was used.

Pregnant women with mental disorders or who were unable to provide informed consent were not enrolled. Potential pregnant women with twin gestations before 12 GW were consecutively enrolled by trained investigators at registration in hospitals during study period. In this study, eligible criteria of twin pregnant women included (a) receiving prenatal health care and admitting to delivery at the tertiary hospital, (b) having examined hsCRP in early pregnancy, (c) delivering at least one live birth. Women with severe pathological conditions, such as Twin-Twin Transfusion Syndrome or Twin Reversed Arterial Perfusion related to monochorionic pregnancies were excluded.

At enrollment, we collected information including maternal age, education level (middle school or less, high school, postgraduate or above), couple income per year (< 12, 12 ~ , 20 ~ , 30 ~ ten thousand yuan), prepregnant BMI (< 18.5, 18.5 ~ , 24 ~ kg/m^2^), parity (0,1 ~), active or passive smoke, alcohol, date of last menstrual period (LMP), anti-inflammatory drugs use, progestogens use, type of conception (spontaneous, assisted reproductive technology (ART)), type of chorionic membrane, vaginosis or periodontitis. Threatened premature labor (TPL) and PROM were obtained in both the second and third trimesters. Spontaneous abortion, stillbirth, and fetal reduction operation were also assessed as fetal loss before delivery. After enrollment, women were followed up at 24–28 GW, 28–32 GW, and during delivery in the hospitals.

### Sample collection and hsCRP measurement

The blood samples from women were drawn during 5–18 GW and serum was separated and examined within 2 h after collection. All standardized procedures were conducted by clinical laboratory staffs in the tertiary hospital. Maternal serum hsCRP concentrations were determined by a validated high-sensitivity particle-enhanced immunoturbidimetric assay (DiaSys Diagnostic Systems, Holzheim, Germany) on the Beckman-Coulter AU5800 analyzer (CA, USA). Coefficients of variation were 2.6% (intraassay) and 1.0% (interassay), with a limit of detection (LOD) as 0.05 mg/L. The concentration of hsCRP (3 of those assayed) below the LOD were imputed using the half LOD (0.025 mg/L). The outlier of hsCRP was identified as a value > 3 IQRs from the 25th or 75th percentiles.

### PTD definition

The primary outcome was overall PTD, which was defined as delivery before 37 GW [17]. PTD was further divided into sPTD and mPTD. mPTD was defined as PTD simultaneously with preeclampsia (diagnosed by professional obstetricians with maternal hypertension and/or proteinuria), fetal stress (at least one of twin was evaluated by professional obstetricians mainly with antenatal cardiotocography), fetal growth restriction (at least one of twin was diagnosed by professional ultrasonologist), or antenatal hemorrhage (mainly caused by placenta previa or placental abruption) [18]. The remaining of overall PTD was defined as sPTD, including PTD initiated by spontaneous labor or preterm premature rupture of membranes. For monochorionic pregnancies, elective cesarean preterm deliveries without medical necessity were also considered as sPTD.

### Statistical analyses

Each twin-pregnant woman rather than every fetus was the study unit. Using the Shapiro–Wilk Normality Test, serum hsCRP had non-normal distribution (*P* < 0.001), with a skewness of 7.28 and a kurtosis of 77.97. Thus, geometric mean (GM) was used to describe serum hsCRP. To calculate GM, hsCRP was log transformed and then back transformed into the initial scale. GM (95% CI) or frequency (percent) were used to characterize descriptive data. Kruskal–Wallis test and post hoc Wilcoxon tests with the Bonferroni method for multiple comparisons were used to examine the discrepancies for continuous variables. Chi-square test and multiple comparisons with the Bonferroni method were conducted for testing the differences of categorical variables.

Univariable and multivariable linear regression models on logarithm transformed hsCRP were conducted to estimate unadjusted and adjusted least squares GM. Mann–Whitney rank sum test was used to compare the GMs between PTDs and term delivery.

When association analysis was performed, hsCRP, as exposure, was conducted as a tertile variable because the highest tertile was close to the abnormal range clinically. Univariable and multivariable logistic regression models were used to calculate unadjusted and adjusted odds ratio [OR] and 95%CI for PTDs among hsCRP tertiles, with the lowest tertile serving as the reference. Because of the high PTD rates among twin gestations, ORs were subsequently converted to relative risks [RR] [19]. In multivariable models, covariates included maternal age, couple income per year, prepregnant BMI, GW at blood drawing, and type of conception were adjusted.

To determine the robustness of the association between hsCRP and PTDs, subgroup analyses stratified by baseline characteristics were performed. In addition, sensitivity analyses were performed by excluding women with vaginosis (*n* = 12) or periodontitis (*n* = 16) at enrollment, concerning that vaginosis [20] or periodontitis [21] may contribute to sPTD.

Based on the cohort study design, it was estimated that a sample of 281 twin-pregnant women would have a 90% power to identify the significant association between hsCRP and sPTD equal to or higher than 1.3, considering a 30% incidence rate among twin gestations.

All analyses were conducted using R software version 4.0.2, and all *P* values were two sided with the statistical significance level of 0.05.

## Results

A total of 618 twin gestations were included in the study, after excluding one woman with outlier of hsCRP. The GMs of hsCRP tertiles were 0.47 (95%CI: 0.42–0.53), 1.99 (95%CI: 1.91–2.06) and 6.13 (95%CI: 5.69–6.61), respectively. Demographic and maternal characteristics of all participants are summarized by hsCRP tertiles in Table 1. Pregnant women in the highest tertile of hsCRP were more likely to be older (*P* = 0.011), using progestogens at baseline (*P* = 0.004), conceiving by ART (*P* = 0.008), having a higher GW at blood drawing (*P* = 0.005) and a lower educational level (*P* < 0.001), compared with those in the lowest tertile. Women with higher hsCRP levels could be seen in those whose prepregnant BMI were greater than 24 kg/m^2^ (44.61%, 20.10%, 11.71%; K-W test: *P* < 0.001), and pairwise comparisons were all statistically significant.

**Table 1:** Demographic and maternal characteristics of participants by hsCRP tertiles

	N	Tertile 1 (N, %)	Tertile 2 (N, %)	Tertile 3 (N, %)	*P*
**N**	618	205(33.2)	209(33.8)	204(33.0)	
**hsCRP in early pregnancy(mg/L)**	618	0.47(0.42,0.53)	1.99(1.91,2.06)	6.13(5.69,6.61)	
**Maternal age(year)**	618	32.61(32.04,33.19)	33.28(32.80,33.77)	33.67(33.12,34.23)^ac^	0.011
**Education level**					< 0.001
Middle school or less	64	12(5.9)	18(8.6)	34(16.7)^ac,bc^	
High school	348	107(52.2)	114(54.6)	127(62.2)	
Postgraduate or above	206	86(41.9)	77(36.8)	43(21.1)	
**Couple income per year** **(10,000yuan)**					0.031
< 12	156	43(21.0)	47(22.5)	66(32.3)	
12 ~	115	44(21.5)	35(16.7)	36(17.7)	
20 ~	148	48(23.4)	48(23.0)	52(25.5)	
30 ~	199	70(34.1)	79(37.8)	50(24.5)	
**Prepregnant BMI (kg/m**^**2**^**)**					< 0.001
< 18.5	39	25(12.2)	10(4.8)	4(2.0)^ab,ac,bc^	
18.5 ~	422	156(76.1)	157(75.1)	109(53.4)	
24 ~	157	24(11.7)	42(20.1)	91(44.6)	
**Parity**					0.573
0	527	179(87.3)	175(83.7)	173(84.8)	
1 ~	91	26(12.7)	34(16.3)	31(15.2)	
**Active or passive smoke**					0.733
No	501	165(80.5)	173(82.8)	163(79.9)	
Yes	117	40(19.5)	36(17.2)	41(20.1)	
**Alcohol**					0.367
No	552	178(86.8)	189(90.4)	185(90.7)	
Yes	66	27(13.2)	20(9.6)	19(9.3)	
**Anti-inflammatory drugs use**					0.405
No	455	145(70.7)	160(76.6)	150(73.5)	
Yes	163	60(29.3)	49(23.4)	54(26.5)	
**Progestogens use**					0.004
No	142	60(29.4)	50(24.0)	32(15.8)^ac^	
Yes	473	144(70.6)	158(76.0)	171(84.2)	
**Type of conception**					0.008
Spontaneous	180	72(35.3)	64(30.6)	44(21.6)^ac^	
Assisted reproductive technology	437	132(64.7)	145(69.4)	160(78.4)	
**Type of chorionic membrane**					0.347
Dichorion	455	144(70.6)	155(74.5)	156(76.9)	
Monochorion	160	60(29.4)	53(25.5)	47(23.1)	
**Gestational weeks at blood drawing**	618	8.56(8.31,8.81)	8.94(8.69,9.19)	9.16(8.89,9.43)^ac^	0.005
**Threatened premature labor**					0.999
No	476	158(77.1)	161(77.0)	157(77.0)	
Yes	142	47(22.9)	48(23.0)	47(23.0)	
**Premature rupture of membrane**					0.342
No	506	165(80.9)	177(85.9)	164(81.6)	
Yes	105	39(19.1)	29(14.1)	37(18.4)	
**Fetal loss**					0.367
No	526	178(86.8)	172(82.3)	176(86.3)	
Yes	92	27(13.2)	37(17.7)	28(13.7)	

Continuous variables: GM (95%CI), K-W test. Categorical variables: N (%), chi square test

^ab, ac, bc^expresses the result of pairwise comparison among hsCRP tertiles and the significant level was adjusted by Bonferroni, for instance, ac shows the significant difference of T1 and T3

The range of gestational age at delivery was 26–41.5 GW, with 302 women delivered at preterm, among which 166 (54.97%) were sPTD and 136 (45.03%) were mPTD. The comparisons of crude and adjusted least squares GMs for hsCRP between PTDs and term deliveries are presented in Table 2. The crude least squares GMs for hsCRP in overall PTD (2.12 mg/L), sPTD (1.94 mg/L) and mPTD (2.22 mg/L) were consistently higher than that in term deliveries (1.51 mg/L) (all *P* < 0.001). After multivariable adjustment, only sPTD had a greater hsCRP levels compared to term delivery (2.20 mg/L vs. 1.84 mg/L,* P* < 0.001).

**Table 2:** Geometric means (GM) [95% CI] of hsCRP among different delivery status

	N(%)	Crude hsCRP (mg/L)			Adjusted hsCRP (mg/L)		
		GM	(95%CI)	*P*	GM	(95%CI)	*P*
Term delivery	316(51.13)	1.51	(1.45,1.52)	(reference)	1.84	(1.80,1.88)	(reference)
Overall PTD	302(48.87)	2.12	(2.11,2.13)	< 0.001	2.13	(2.09,2.16)	< 0.001
sPTD	166(54.97)	1.94	(1.93,1.95)	< 0.001	2.20	(2.15,2.24)	< 0.001
mPTD	136(45.03)	2.22	(2.20,2.24)	< 0.001	1.84	(1.80,1.87)	0.560

Linear regression calculates logarithm transformed hsCRP, and least squares GMs are retransformed to the original scale

Mann–Whitney rank sum test for difference in least squares GMs by delivery status

Adjusted for maternal age, couple income per year, prepregnant BMI, GW at blood drawing, type of conception

The crude and adjusted relative risk (RR) of PTDs among hsCRP tertiles referring to the lowest tertile are illustrated in Table 3. In the crude analyses, women in the highest tertile of hsCRP, had increased risks of overall PTD (RR 1.40, 95%CI: 1.10–1.74), sPTD (RR 1.36, 95%CI: 1.00–1.75), and mPTD (RR 1.46, 95%CI: 1.07–1.89), compared with those in the lowest tertile. After adjustment of maternal age, couple income per year, prepregnant BMI, GW at blood drawing and type of conception, women in the highest tertile of hsCRP still presented significant higher risks for developing overall PTD (adjusted relative risks [ARR] 1.42, 95%CI: 1.08–1.78) and sPTD (ARR 1.49, 95%CI: 1.08–1.93), but not for mPTD (ARR 1.32, 95%CI: 0.91–1.79).

**Table 3:** Relative risk of PTDs in association with hsCRP tertiles

	N(%)	Crude RR (95%CI)				Adjusted RR (95%CI)			
		Tertile 1	Tertile 2	Tertile 3	*P*_trend_	Tertile 1	Tertile 2	Tertile 3	*P*_trend_
**hsCRP Median (IQR)**	618(100)	0.58 (0.31–0.94)	1.91 (1.61–2.49)	5.56 (4.16–7.66)		0.58 (0.31–0.94)	1.91 (1.61–2.49)	5.56 (4.16–7.66)	
**Overall PTD**	302(48.87)	1(reference)	1.19 (0.91,1.51)	1.40** (1.10,1.74)	0.032	1(reference)	1.23 (0.93,1.56)	1.42** (1.08,1.78)	0.047
**sPTD**	166(26.86)	1(reference)	1.18 (0.86,1.55)	1.36* (1.00,1.75)	0.148	1(reference)	1.25 (0.90,1.66)	1.49* (1.08,1.93)	0.060
**mPTD**	136(22.01)	1(reference)	1.20 (0.84,1.62)	1.46* (1.07,1.89)	0.063	1(reference)	1.19 (0.83,1.62)	1.32 (0.91,1.79)	0.309

Adjusted for maternal age, couple income per year, prepregnant BMI, GW at blood drawing, type of conception

There was a subtle trend between hsCRP tertiles and sPTD, close to statistical significance (*P*_trend_ = 0.060) after adjusting for covariates. To note, removing the serum samples of below LOD (*n* = 3), the linear trend between hsCRP tertiles and sPTD turned to be significant (*P*_trend_ = 0.046), with the 1.51-fold risk (95% CI: 1.10,1.96).

**Fig. 1:**
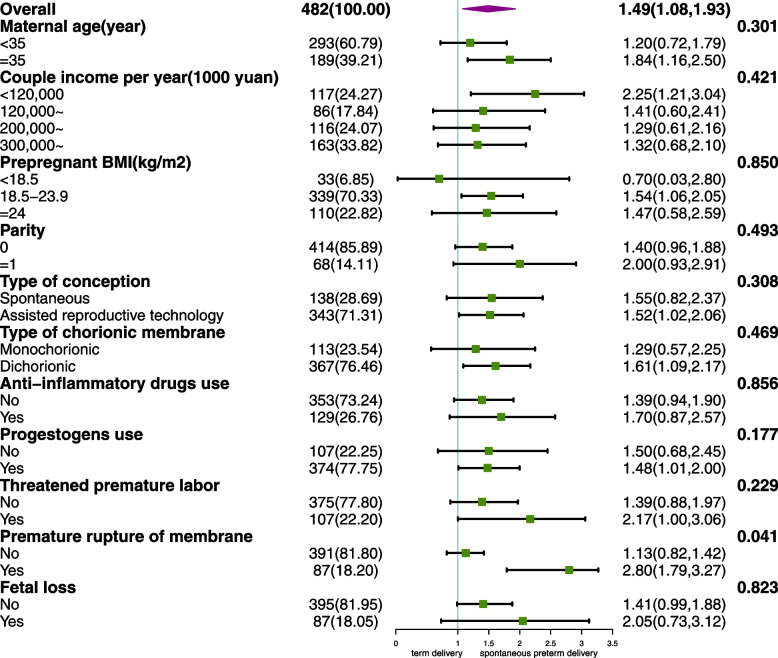
Subgroup analyses of hsCRP tertiles and sPTD

To confirm the robustness of the hsCRP-sPTD association, subgroup analyses were performed (Fig. 1). The increased risk of sPTD in the highest tertile of hsCRP was consistently observed in some subgroups. Moreover, a significant interaction between hsCRP tertiles and PROM was found (*P* value for interaction test = 0.041), and no statistical interactions were observed for other covariates.

Sensitivity analyses were performed to examine the confounding effects on RRs by excluding women who had vaginosis or periodontitis in early pregnancy. There was almost no change in the results: after excluding women who had vaginosis alone (ARR = 1.50, 95%CI: 1.09–1.95) or periodontitis alone (ARR = 1.45, 95%CI: 1.03–1.89), the top tertile of hsCRP still increased the risk of developing sPTD; and further excluding both, the ARR was 1.45 (95%CI: 1.04–1.97).

## Discussion

From this prospective cohort study, we found that elevated hsCRP level in early pregnancy is positively associated with sPTD rather than mPTD among Chinese twin gestations. Compared with the lowest hsCRP tertile (lower than 1.26 mg/L), the highest hsCRP tertile (higher than 3.22 mg/L) increased almost 1.5-fold risk of sPTD, whether there existed vaginosis or periodontitis in early pregnancy or not. We also found that PROM appeared to be a strong positive modifier of this relationship. To the best of our knowledge, this is the first prospective cohort study specifically for twin gestations to examine the relationship of hsCRP in early pregnancy and PTD, which includes both sPTD and mPTD. Our findings fill the gap for twin gestations to support the hypothesis that early pregnancy inflammation contributes to sPTD, which has been reported in singleton pregnancies [11–13, 22, 23].

There are no uniform standards for normal hsCRP range for pregnant women. Due to various detection assays and mixed populations, the references differed by hospitals. In this tertiary hospital, hsCRP ≥ 3 mg/L was defined as out of normal range. The highest tertile hsCRP was > 3.22 mg/L in our study, close to the reference range. Up to date, we had not found any research on the levels of hsCRP in twin pregnancies. Studies in singleton pregnancies showed that the levels of CRP increased in the first trimester, peaked at about 20 weeks, and then decreased gradually until delivery [24]. Those serum CRP was measured by ELISA, which could not deal as well with CRP out of standard curve as the particle-enhanced immunoturbidometric method could. Assay method in our study is more sensitive, making it possible to test a lower LOD of hsCRP. Also, the median GWs of serum detection in previous studies were later than ours, as hsCRP is known to increase with increasing GW [24].

The incidence of PTD among twin gestations (48.9%) was consistent with that reported in other comparable studies in China [5] or other countries [18, 25]. In addition, the proportion of PTD subtypes in our study (55.0% sPTD and 45.0% mPTD) was also close with previous studies [5, 25]. To note, we distinguished sPTD from overall PTD. Pregnant women with medical indications who underwent an elective cesarean delivery before 37 GW were considered as mPTD [5, 25]. The incidence of PTD differed by types of chorionic membrane in twin-pregnancy. Monochorionic twins had a higher incidence of PTD than dichorionic twins [26], which was consistently observed in our study (70.0% vs 41.32%, *P* < 0.001).

The magnitude of the hsCRP-sPTD association in our study (ARR 1.5) was weaker than that in a singleton pregnancy study [11] (serum hsCRP cut-off values ranged from 5.6 mg/L to 7.6 mg/L; corresponding ORs ranged from 1.7 to 2.0). A cohort study from Iran conducted in 120 singleton gravidas [13] also showed that one-unit increase in early serum hsCRP increased the risk of sPTD by 2.3-fold. And other two singleton pregnancy studies even had stronger significant ORs on the hsCRP concentration over 8 mg/L (ORs: 2.6 and 2.9) [12, 22]. The positive hsCRP- sPTD association was consistently observed across various subgroups. The association was only observed in dichorionic twins but not in monochorionic twins probably because the latter were more likely to be electively induced before or at 36 GW. In addition, a majority of lower limits of 95% CIs for significant RRs in subgroup analyses were ranging from 1.01 to 1.19, indicating that the effect might be mild. However, it is worth noting that even the modest association may still be of considerable public health importance given the extremely high rates of PTD among twin gestations.

Biological plausibility for the adverse effect of hsCRP on sPTD is based on the relationship between inflammatory response and labor process [7, 27–29]. The current clinical consensus is that inflammation in early pregnant may result in sPTD in singleton gravida, while the most widely accepted explanation for twin gestations is uterine overdistention [7]. A recent experiment suggested that inflammation may response to uterine overdistention, which was also observed in amnion and myometrium of women with twins in vitro [30]. The evidence supported the potential relationship between inflammation and sPTD among twin gestations. On the one hand, pro-inflammatory cytokines such as IL-1, IL-6, IL-8 and tumor necrosis factor α (TNF-α) activate the uterus by stimulating prostaglandin synthesis and decreasing the response of tissue progesterone [7]; through the regulation of pro-inflammatory cytokines, hepatocytes secrete CRP in response to inflammation and then release into the systemic circulation. On the other hand, hsCRP appeared an increasing trend in early pregnancy [24], and the inflation of uterus also increased with GW. Thus, elevated hsCRP may be a potential accompanying process for uterine overdistention among twin gestations.

We did not observe any association between hsCRP and mPTD in overall and subgroup analyses. There was a dramatic difference between crude and adjusted RRs for hsCRP-mPTD association (positive to negative). In the full-adjusted models, we found that removing BMI categories made the ARR (1.45, 95% CI: 1.04–1.90) significantly stronger than that non-significant one (1.33, 95% CI: 0.91–1.79), indicating that prepregnant BMI was a potential confounding factor, attenuating the association between hsCRP and mPTD. Previous study found that overweight or obese women were more likely to trigger the hsCRP-PTD pathway [31], supporting our findings. We did not observe the association in this population, probably due to the limited sample size (only 110 women had prepregnant BMI ≥ 24 kg/m^2^) after stratified by PTD subtypes.

Our study has several strengths. First, well-implemented cohort follow-up visits enabled us to obtain abundant reliable information on covariates, and detailed diagnosis information made it possible for us to distinguish mPTD from sPTD. Second, replication of the association in different demographic and pregnant character settings, and in sensitive analyses enhanced the validity of results. Third, we found that PROM had a modification effect between hsCRP-sPTD association. The simultaneous presence of early elevated hsCRP and later PROM multiplied the risk of sPTD (from 1.49 to 2.80), consisting that PROM women may exist subclinical inflammation [32]. Finally, to avoid diagnostic suspicion bias, we further analyzed the incidence rate of PTD between women with and without hsCRP values, and the post hoc analysis showed that it was a random event for women to check hsCRP or not (PTD rates: 49.0% vs 55.6%, *P* = 0.210).

There are still some limitations in our study. There are 85.2% of participants were nulliparous women, thus caution is needed when generalizing our results to multiparous women. Among multiparous women in our study, there was only one had had a previous PTD, limiting us to assess the effect of previous PTD history on our results. In addition, each twin-pregnant woman in our study measured hsCRP mainly during first trimester, but there are no hsCRP measurements during second trimester or third trimester. Thus, we cannot examine hsCRP trend in sPTD risks throughout the entire pregnancy. Besides, information about vaginosis or periodontist was self-reported and information on cervical length status at baseline was not collected, which might introduce potential bias to our results.

## Conclusion

To summarize, hsCRP was positively associated with an increased risk of sPTD among twin gestations. Our findings indicated that inflammation in early pregnancy might influence the timing of labor onset, leading sPTD. Future studies are needed to investigate the effect of a spectrum of inflammatory markers from different trimesters on the risk of PTD among twin gestations.

## Data Availability

The datasets generated during and analysed during the current study are not publicly available due to the personal privacy and the hospital policy, but are available from the corresponding author on reasonable request.

## Abbreviations

PTD: Preterm delivery
sPTD: Spontaneous preterm delivery
mPTD: Medical-induced preterm delivery
hsCRP: High-sensitivity C- reactive protein
GW: Gestational week
ART: Assisted reproductive technology
TPL: Threatened premature labor
PROM: Premature rupture of membrane
GM: Geometric means
RR: Relative risk
ARR: Adjusted relative risk
BMI: Body mass index
